# Migration Rules and Mechanisms of Nano-Biochar in Soil Columns under Various Transport Conditions

**DOI:** 10.3390/nano14121035

**Published:** 2024-06-15

**Authors:** Peng Li, Meifang Yan, Min Li, Tao Zhou, Huijie Li, Bingcheng Si

**Affiliations:** 1 College of Resources and Environmental Engineering, Ludong University, Yantai 264025, China; lipeng530@163.com (P.L.); bing.si@usask.ca (B.S.); 2 Key Laboratory of Agricultural Soil and Water Engineering in Arid and Semiarid Areas, Northwest A&F University, Xianyang 712100, China; ymf@nwafu.edu.cn (M.Y.); limin2016@nwafu.edu.cn (M.L.); 3 Department of Soil Science, University of Saskatchewan, Saskatoon, SK S7N 5A8, Canada

**Keywords:** nano-biochar, nanoparticle, soil columns, migration rules, penetration curves

## Abstract

Compared to traditional biochar (BC), nano-biochar (NBC) boasts superior physicochemical properties, promising extensive applications in agriculture, ecological environments, and beyond. Due to its strong adsorption and migration properties, NBC may carry nutrients or pollutants to deeper soil layers or even groundwater, causing serious environmental risks. Nevertheless, the migration rules and mechanisms of NBC in soil are still unclear. Therefore, this study employed soil column migration experiments to systematically explore the migration rules and mechanisms of NBC under various flow rates, initial soil water contents, soil depths, and soil textures. The results showed that regulated by smaller particle size differences and greater surface charges, NBC exhibited a stronger migration ability compared with traditional BC. As the soil texture transitioned from fine to coarse, the migration capability of NBC significantly improved, driven by both pore structure and interaction forces as described by the DLVO theory. The migration ability of NBC was also greatly boosted as the soil transitioned from saturated to unsaturated conditions, primarily because of preferential flow. When the flow rate increased from 70% KS to 100% KS and 130% KS, the migration ability of NBC also increased accordingly, as changes in injection flow rates altered the velocity distribution of pore water. NBC in 25 cm soil columns was more prone to shallow retention compared with 10 cm soil columns, resulting in weaker overall migration ability. In addition, through fitting of the two-site kinetic model and related parameters, the penetration curves of NBC under various variable conditions were effectively characterized. These findings could offer valuable insights for NBC’s future efficient, rational, and sustainable utilization, facilitating the evaluation and mitigation of its potential environmental risks.

## 1. Introduction

Nano-biochar (NBC) is a highly aromatic, carbon-rich nanoparticle obtained from biomass materials such as agricultural waste, municipal waste, and animal manure with a high carbon content [1]. It is first converted into biochar (BC) under limited oxygen conditions at relatively low temperatures (300–700 °C), and then several modification scenarios, including chemical treatment, steaming, gaseous activation, natural aging, and ball milling, are suggested for the refinement of BC into NBC [2,3]. The size of NBC typically ranges from 10 to 100 nanometers, and it is black in color [4]. Compared to traditional BC, NBC has a series of superior physicochemical properties such as large specific surface area, high micropore rate, strong ion exchange capacity, rich surface functional groups, high stability, and strong adsorption capacity [5,6,7,8]. These attributes confer upon NBC distinct advantages in enhancing soil’s capacity to retain water and fertilizers, mitigating the effects of heavy metal contamination, and ameliorating soil structure. NBC thus emerges as a promising tool for advancing ecological environments and unlocking the agricultural sector’s potential for sustainable applications [9,10,11]. Previous studies have shown that after the preparation of BC using different raw materials and pyrolysis temperatures through the oxidation process or different ball milling parameter treatment, compared with non-oxidized BC, the oxygen-containing functional groups on the surface of oxidized BC increase significantly by 3.9–53.4%. Furthermore, there have been significant enhancements in specific surface area, pore volume, and cation exchange capacity [12,13]. Currently, NBC has become a material that receives more attention than BC [4,14]. In general, the morphology and structure of NBC can be controlled by selecting different catalysts, solvents, and temperatures to meet specific application requirements [2,3,4].

Due to the powerful adsorption function of BC, coupled with the strong migration ability of nanoparticles themselves [15,16], the migration of NBC, which combines the characteristics of both, in porous media has attracted much attention. Previous studies have shown that NBC may transport adsorbed nutrients and even pollutants to deeper soil layers or even groundwater through runoff, infiltration, and other routes [6,17,18]. This process can expedite the leaching of nutrients and the dispersion of pollutants, consequently exacerbating environmental risks [19,20,21]. Therefore, exploring the migration rules and mechanisms of NBC in soil is crucial for fully leveraging its advantages and reducing potential negative impacts.

Previous studies on the transport of BC and NBC have shown that the migration of nanoparticles in porous media is influenced by their own characteristics, the chemical conditions of the carrier matrix, and the hydraulic characteristics of the porous media. In terms of the characteristics of BC nanoparticles, smaller particles are more prone to migration and have lower attachment efficiency in porous media, while larger particles are more likely to experience exclusion effects in porous media [22,23]. Wang et al. conducted migration experiments using wheat straw BC with different pyrolysis temperatures (350, 550 °C) in quartz sand columns. The results showed that NBC pyrolyzed at lower temperatures has more hydrophilic surface functional groups and a stronger migration ability compared to NBC pyrolyzed at higher temperatures [24].

In terms of the chemical conditions of the carrier matrix, previous studies have shown that high ionic strength can compress the electric double layer of nanoparticles, weakening the repulsive force and promoting aggregation. Chen et al. found that by suddenly reducing the ionic strength of the carrier solution by five times (from 50 mM to 10 mM), a large number of BC colloidal particles retained on the surface of soil particles were released [25]. Additionally, the type of ions also affects the migration properties of NBC: divalent cations (Ca^2+^) exhibit greater efficacy in inhibiting the migration of NBC in soil than monovalent cations (Na^+^) [26]. This is mainly due to the fact that higher-valent cations are more likely to form bridges with NBC, leading to NBC aggregation. When the pH of the carrier matrix approaches the zero charge point, i.e., the pH at which the surface charge of NBC tends to zero, the repulsion between nanoparticles and porous media decreases, promoting their aggregation [27], resulting in the reduced migration ability of NBC.

In terms of the characteristics of porous media, the migration of NBC in unsaturated soil is greatly influenced by soil water content. Liu et al. found that the migration ability of NBC in unsaturated quartz sand columns is lower than that in saturated quartz sand columns [28]. Furthermore, the presence of NBC in small pores with strong adsorption forces can also lead to increased adsorption and reduced mobility [29,30]. Additionally, the flow velocity of pore water can also alter its mobility [23,31]. For example, some scholars have controlled the flow velocity of ball-milled NBC in quartz sand columns and found that the migration rate of BC is extremely high at high flow velocities, with the time to penetrate the sand column reduced by multiples [6,32].

In addition to qualitative analysis, many scholars currently use the classic DLVO theory and two-site kinetic model to semi-quantitatively explain the migration rules and mechanisms of NBC in porous media. The DLVO theory divides the interaction forces between nanoparticles and porous media into van der Waals forces and electric double layer forces [33,34]. According to this theory, when the attractive force is greater than the repulsive force, particles tend to aggregate, and conversely, the particle system tends to disperse. The greater the dispersion of the system, the more pronounced the migration of nanoparticles and the lower the retention in porous media. The two-site kinetic model is a partial differential equation system constructed based on mass balance and solute transport mechanisms, and predicts the spatial and temporal changes in nanoparticle concentration or flux in soil through analytical or numerical solutions [35]. Such models consider both the mobility of soil pore water and the heterogeneity of soil particle adsorption of nanoparticles, which best reflect the migration process of nanoparticles in soil. However, the two-site kinetic model has many parameters, most of which cannot be directly measured. Currently, the most commonly used indirect estimation method is to use measured data combined with the least squares method to inversely solve the model parameters [36].

To date, there are still some limitations in the current research on the transport of BC and its nanoparticles. Firstly, previous studies have mainly focused on bulk BC, micron-sized BC, or colloidal BC with hydrodynamic diameters ranging from 100 nm to 1 μm. There is a scarcity of research on the transport mechanisms of carbon nanoparticles with hydrodynamic diameters smaller than 100 nm [37,38]. Secondly, most scholars usually use indoor quartz sand columns to explore the migration capacity of NBC [38,39,40]. The complexity and heterogeneity of natural soil far exceed the scope that can be simulated by quartz sand, which possesses single physicochemical characteristics [25]. Concerning this issue, some scholars have used small soil columns (1.2 × 6 cm) to study the migration patterns and mechanisms of NBC in carrier flow media under different chemical conditions [25]. However, due to the complexity of particle migration in soil [41], such small soil columns cannot fully reflect migration behavior at a large scale. Thirdly, there remains a limited understanding of how the characteristics of nanoparticles in soil and porous media influence their migration.

This study takes NBC prepared from the main agricultural and forestry waste in the Loess Plateau, with apple branches as the main research object, and explores the migration rules and mechanisms of NBC in the soil as the main research line. By conducting soil column migration experiments and utilizing two-site kinetic models alongside the DLVO theory and Hydrus-1D V4.16 software, this study investigates the migration patterns and influencing mechanisms of NBC in soil under various conditions, including BC particle size, soil texture, injection flow rate, initial soil water content, and soil column depth. The results are expected to establish a theoretical foundation for applying NBC to repair ecological problems in the Loess Plateau. This would enable the enhancement of soil water and fertilizer retention capacity, the improvement of water and fertilizer utilization efficiency and crop yield, and a reduction of pollution in deep soil and groundwater.

## 2. Materials and Methods

### 2.1. Preparation and Characterization of Nano-Biochar from Apple Branches

**Figure 1:**
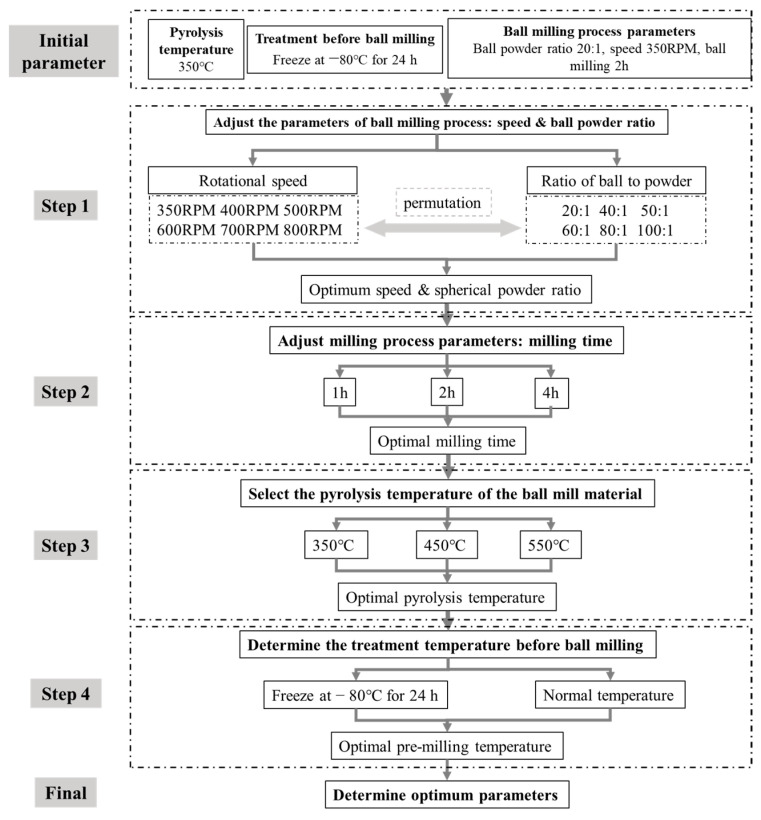
Optimization steps of parameters for the preparation of nano-biochar by ball milling.

**Figure 2:**
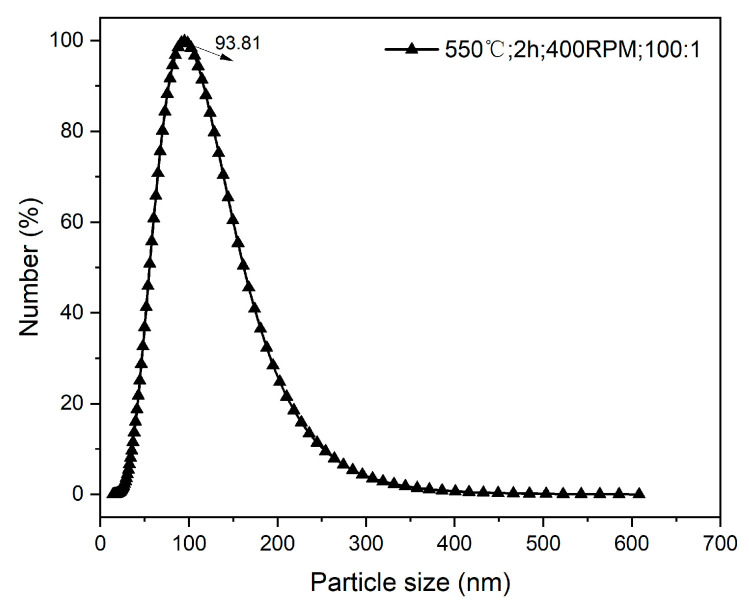
Size distribution of nano-biochar particles.

One to two-year-old branches of apple trees were selected as the material source, and the fruitwood branches were pyrolyzed under limited oxygen conditions to generate bulk biochar. The specific operation steps were as follows: the apple branches were dried at 65 °C for 72 h, crushed with a branch crusher, and then sifted with a 1 mm sieve. The apple branches were then pyrolyzed at 350 °C for 2 h in a muffle furnace under N_2_ atmosphere. After cooling, the branches were crushed and sifted through a 100-mesh sieve to obtain the bulk BC. Taking into account multiple factors such as the ball milling effect, time, cost, and output, the optimal combination of parameters for preparing NBC was determined by gradually optimizing the ball milling parameters. The ball milling parameters were set as follows: the ball-to-powder ratio was 100:1, the rotating speed was 400 RPM, the ball milling time was 2 h, and the pyrolysis temperature was 550 °C with the ball mill grinding for 5 min and then standing for 5 min to prevent particle aggregation caused by temperature rise due to high energy input during the grinding process. The pretreatment temperature had no significant effect on the grinding effect (*p* > 0.05). After ball milling, the product was sieved with a 100-mesh sieve to obtain NBC. The operation steps are shown in Figure 1 and the particle size distribution is shown in Figure 2.

The properties of the prepared BC and NBC, including particle size, specific surface area, pore volume, cation exchange capacity, elemental composition, zeta potential, and morphological characteristics, were characterized. The specific methods were as follows.

To assess the particle size of the bulk BC, we employed the Malvern laser particle size analyzer (Mastersizer 2000, Malvern, UK), following the specific operational steps outlined in the previous literature [2]. However, as the measurement range of the laser particle size analyzer is primarily focused on micrometer levels and above, dynamic light scattering (DLS) (Omni, Brookhaven, New York, NY, USA) technology was further used to precisely measure the nanometer-scale particle size of NBC. Before measurement, the NBC was first prepared into a 100 mg L^−1^ suspension and subjected to ultrasonic treatment for 3 min to ensure uniform dispersion of NBC particles in the aqueous solution. The diameter measured directly by DLS is the hydrodynamic diameter of the particles. This includes both the particle’s own diameter and the thickness of the surrounding double electric layer [14]. For smaller particles, this diameter may not accurately reflect their true size. It is generally acknowledged that the number-weighted diameter provides a closer approximation to the actual size of the particles [25]. Therefore, in this study, we converted the hydrodynamic diameter measured by DLS into a number-weighted diameter to more accurately represent the particle size of NBC.

The porosity analyzer (model ASAP 2460, manufactured by Micromeritics, Shanghai, China) was used to quantify the surface area and pore structure of BC and NBC, and the automatic specific surface area. In this process, the dried samples were first placed in a sealed quartz tube and underwent degassing treatment at a constant temperature. Subsequently, the samples underwent nitrogen adsorption and desorption under specific conditions. The data were then simulated and analyzed using the system’s software, resulting in precise measurements of the biochar’s specific surface area and pore volume.

In assessing the cation exchange capacity (*CEC*) of BC and NBC, the ammonium acetate exchange method was employed. This method was used to effectively evaluate the samples’ adsorption capacity for various cations. Subsequently, a full-spectrum inductively coupled plasma optical emission spectrometer (model ICP 6300, ThermoFisher Scientific, Waltham, MA, USA) was utilized to precisely determine the concentrations of exchangeable potassium, calcium, sodium, magnesium, and aluminum ions in the samples.

Exchangeable cations were calculated as follows:

(1) $$M^{+}cmol\left(+\right)kg^{-1}=Ccmol\left(+\right)L^{-1}\times \left(0.25\;L/wt.g\right)\times 1000\;g\cdot kg^{-1}$$

$M^{+}$ represents the concentration of exchangeable cation, *cmol*(+)kg^−1^; *C* represents the concentration of cation in the ammonium acetate extract, *cmol*(+)L^−1^; and wt. represents the weight of the air-dried soil sample, g.

The *CEC* was calculated as follows:

(2) $$CECcmol\left(+\right)kg^{-1}=\left[mg\times L^{-1}N\times \left(1cmol\left(+\right)/140\;mg\right)\right]\times \left(0.25\;L/wt.g\right)\times 1000\;g\cdot kg^{-1}$$

The elemental contents of carbon (*C*), hydrogen (*H*), and nitrogen (*N*) in BC and NBC were obtained through elemental analysis using an elemental analyzer (ECS 4024, Costech, Italy), while the content of oxygen (*O*) was calculated by the difference method using the following formula:

(3) $$O\left(\%\right)=100\%-ash\left(\%\right)-C\left(\%\right)-H\left(\%\right)-N\left(\%\right)$$

ash represents the ash content of the BC and NBC, and the measurement refers to the charcoal and the test methods for charcoal (GB/T17996-1999) [42].

The zeta potential of BC and NBC was measured using a nanoparticle size and zeta potential analyzer (zeta PALS, Brookhaven, New York, NY, USA). A total of 100 mg of the sample was mixed into 1 L of background solution (1 mM of NaCl) and placed in an ultrasonic cleaner for 1 h to ensure its complete dispersion. A sample was then taken and placed in the zeta potential analyzer for measurement.

To gain deeper insights into the morphological characteristics of BC and NBC, a field emission scanning electron microscope (Nova Nano SEM-450, Fei, Hillsboro, OR, USA) was utilized for observation. After the samples were dried at 105 °C, they underwent gold sputtering and were scanned under an appropriate magnification to capture their surface microstructures.

The surface functional groups of biochar were determined by a Fourier Transform Infrared (FTIR) Spectrometer (Vertex70, Bruker, Ettlingen, Germany). The samples were dried at 105 °C and mixed with KBr reagent in a ratio of 1:100, and then pressed into standard tablets with a tablet press for measurement.

### 2.2. Collection and Measurement of Soil Samples

For the following indoor soil column migration experiments, soils with varying textures were collected. These textures included silty loam, loam, and sandy loam, obtained from the surface layer (0–20 cm) of maize fields in Changwu and Shenmu, Shaanxi. The collected soils underwent natural air-drying, during which impurities were eliminated. Subsequently, they were crushed and passed through a 2 mm aperture sieve before storage. The methods for measuring the basic physicochemical properties of the soil were as follows: the soil texture was determined by the pipette method, the soil bulk density was measured using the core sampler method, the pH value was measured using a pH meter (Five Easy Plus, Mettler-Toledo, Beijing, China) under a solid-to-liquid ratio of 1:20, the saturated hydraulic conductivity (Ks) was determined by the double ring sampler method, and the measurements of cation exchange capacity and zeta potential were consistent with the measurement methods for BC. The inorganic nitrogen content was measured using a flow analyzer (AA3, SEAL, Hannover, Germany).

### 2.3. Preparation and Treatment of Other Materials

In the subsequent soil column migration experiments, a certain amount of clean quartz sand needed to be laid at the bottom and top of the soil column to protect the integrity of the soil column and ensure the correct completion of the experiment. The processing steps for quartz sand were as follows: the quartz sand was washed several times with heavy deionized water, then soaked in a 1 mol L^−1^ dilute hydrochloric acid solution for 24 h, and then transferred to a 1 mol L^−1^ sodium hydroxide solution to soak again for 24 h in order to remove some impurity ions contained in the quartz sand. Finally, the quartz sand was repeatedly washed with heavy deionized water until the supernatant of the quartz sand was clear and free of turbidity. Then, it was dried in an oven at 105 °C and passed through a 60-mesh sieve for subsequent use.

Solution preparation: 1 mM of NaCl and NaBr were prepared separately as the background solution and tracer solution for the soil column migration experiment.

Biochar suspension preparation: NBC that was prepared under the optimal ball milling parameters and the bulk BC under the corresponding pyrolysis temperature were used to prepare suspensions. A certain amount of BC/NBC was weighed and placed in a beaker, dispersed in the background solution, and then transferred to a volumetric flask for volume determination. The suspension was subsequently sonicated for 1 h using an ultrasonic cleaner to ensure thorough dispersion, resulting in a stock solution of 1 g L^−1^ BC and its nanoparticle suspension. Before each experiment, the suspension required an additional hour of sonication to prevent particle aggregation.

### 2.4. Nano-Biochar Soil Column Migration Experiment

The migration experiment was conducted with a PVC column with an inner diameter of 10 cm and a height of 30 cm. The soil column was filled in layers using the measured bulk density, with the following layers from bottom to top: 100-mesh nylon mesh, quartz sand (2 cm), 100-mesh nylon mesh, soil (10 cm), quartz sand (2 cm), and filter paper. The function of the lower layer of quartz sand and nylon mesh was to prevent soil particles from infiltrating and flowing out through the pipeline, while the upper layer of quartz sand and nylon mesh served to prevent the migration solution from damaging the flatness of the soil layer.

**Figure 3:**
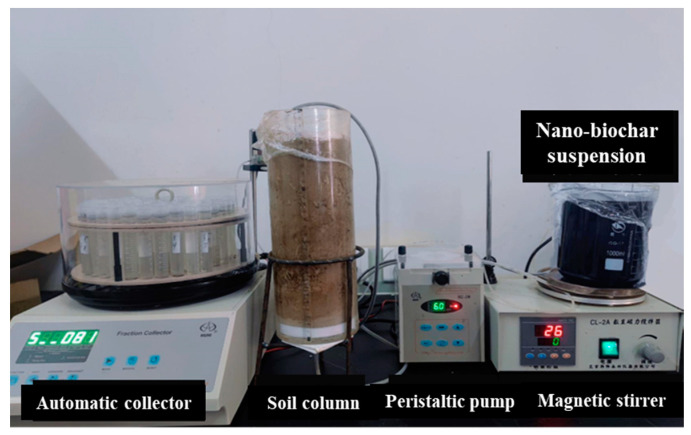
Diagram of soil column migration experiment installation.

A peristaltic pump (HL–2B, Shanghai Yitian Precision Instrument Co., Ltd., Shanghai, China) was used to pump 5 pore volumes (PVs) of background solution into the soil column from bottom to top. The injection flow rate of the peristaltic pump was set to 70% of the measured saturated hydraulic conductivity (Ks) of the soil. After saturating and flushing the packed column, it was left to stand for 24 h to stabilize the internal environment. The weight of the pore water was recorded as the difference between the wet weight and the dry weight of the soil column after saturation, and the pore volume (PV) of the soil column was expressed as the ratio of mass to density. After the completion of the pre-saturation test, 3 PVs of NaBr solution were injected into the soil column from the top at a set flow rate. At least 5 PVs of background solution were injected to flush the column. Samples were collected at fixed time intervals starting from the injection, and the concentration of Br^-^ in the outflow solution was measured using ion chromatography (ICS-1100, ThermoFisher Scientific, USA). The hydrodynamic dispersion coefficient of the soil column was calculated by fitting the breakthrough curve using Hydrus-1D (Figure 3).

After the tracer test, the soil column migration experiments were conducted with different settings for BC particle size (BC/NBC), soil texture (sandy loam/loam/silt loam), injection flow rate (70%/100%/130% of soil saturated hydraulic conductivity), initial soil water content (saturation/field capacity), and soil column length (10 cm/25 cm). The layout of the column experiments is outlined in Table 1. A peristaltic pump was used to pump 3 PVs of BC suspension into the soil column from top to bottom. After the injection of the suspension, at least 5 PVs of background solution were pumped in to flush the soil column until no NBC concentration could be detected in the outflow solution. A magnetic stirrer was used to vigorously stir the suspension during the NBC injection process to prevent aggregation and sedimentation of NBC. The outflow solution was continuously collected at fixed time intervals using a fixed flow rate method through an automatic collector (BS–100A, Analytical Instrument Factory Co., Ltd., Shanghai, China). The NBC concentration in the outflow solution was measured using a UV spectrophotometer (UV2450, Shimadzu Co., Ltd., Kyoto, Japan) at a wavelength of 790 nm, and the penetration curve of NBC particles was plotted based on the measurements. The mass recovery rate of NBC in the outflow solution was obtained based on the mass balance method as the ratio of the carbon content in the outflow solution to the injection amount.

### 2.5. Theoretical Model

(1) DLVO theory

To comprehensively explore the mechanism of NBC migration in soils of different textures, the DLVO theory was used to quantitatively describe the interaction energy between NBC and soil particles, revealing the stability of the particle system. The DLVO theory divides the forces between carbon nanoparticles and porous media into van der Waals attraction (intermolecular forces) and double-layer repulsion. The formula for calculating the total DLVO interaction energy was as follows:

(4) $$\Phi_{\mathrm{Total}}\left(h\right)=\Phi_{EDL}\left(h\right)+\Phi_{vdW}\left(h\right)$$

where $\Phi_{Total}$, $\Phi_{EDL}$, and $\Phi_{vdW}$ represent the total interaction potential energy, the electrostatic potential energy of the electric double layer, and the van der Waals attraction potential energy, respectively; h represents the distance between the carbon nanoparticles and the porous media.

(5) $$\Phi_{EDL}\left(h\right)=\pi \cdot \varepsilon_{0}\cdot \varepsilon_{r}\cdot a_{p}\left\{2\psi_{1}\cdot \psi_{2}\cdot \ln\left[\frac{1+\exp\left(-k\cdot h\right)}{1-\exp\left(-k\cdot h\right)}\right]+\left(\psi_{1}^{2}+\psi_{2}^{2}\right)\ln\left[1-\exp\left(-2k\cdot h\right)\right]\right\}$$

where $\varepsilon_{0}$ is the vacuum permittivity (8.854 × 10^−12^ F m^−1^); $\varepsilon_{r}$ is the relative permittivity (78.5); $a_{p}$ is the hydraulic radius of the carbon nanoparticles; $\psi_{1}$ and $\psi_{2}$ are the Zeta potentials of the carbon nanoparticles and the porous media, respectively; and k is the Debye parameter.

(6) $$k^{2}=\frac{1000e^{2}N_{A}}{\varepsilon_{0}\varepsilon_{r}k_{B}T}\sum_{i}M_{i}{z_{i}}^{2}$$

where $k_{B}$ is the Boltzmann constant (1.38 × 10^−23^ J K^−1^); T is the relative thermodynamic temperature (298 K); e is the charge number of an electron (−1.602 × 10^−19^ C); $N_{A}$ is Avogadro’s constant (6.02 × 10^23^ mol^−1^); $M_{i}$ is the concentration of electrolytes under different environmental conditions; and $z_{i}$ represents the valence state of electrolytes under the same conditions.

(7) $$\Phi_{vdW}\left(h\right)=-\frac{A_{132}}{6}\left(\frac{2a\left(H+A\right)}{H\left(H+2a\right)}+\ln\left(\frac{H}{H+2a}\right)\right)$$

where $A_{132}$ is the Hamaker constant of the biochar nanoparticle–water–soil system; a is the particle radius; and H is the distance between different sites on the surface of the layer and the sphere.

(8) $$A_{132}=\left(\sqrt{A_{11}}-\sqrt{A_{33}}\right)\left(\sqrt{A_{22}}-\sqrt{A_{33}}\right)$$

where $A_{11}$ is the Hamaker constant for NBC particles (4.85 × 10^−20^ J); $A_{22}$ is the Hamaker constant for soil particles (6.5 × 10^−20^ J); and $A_{33}$ is the Hamaker constant for water (3.7 × 10^−20^ J).

(2) Two-point kinetic model

To simulate the migration behavior of NBC in soil and further analyze its migration patterns in soil, the two-point kinetic model based on the one-dimensional convection-dispersion equation was selected to fit the penetration curve of NBC particles. The two-point kinetic model assumes that there are two kinetic adsorption sites for nanoparticles in the medium. One site (P_1_) is a reversible retention site, where BC particles undergo both adsorption and desorption processes in soil. The other site (P_2_) is an irreversible attachment site (exclusion), where only the adsorption process of NBC is considered. The formulas for the two-point kinetic model are outlined below:

(9) $$\frac{\partial \theta C}{\partial t}+\rho_{b}\frac{\partial \left(S_{1}\right)}{\partial t}+\rho_{b}\frac{\partial \left(S_{2}\right)}{\partial t}=\frac{\partial }{\partial x}\left(\theta D\frac{\partial C}{\partial x}\right)-\frac{\partial qC}{\partial x}$$

(10) $$S_{1}:\rho_{b}\frac{\partial \left(S_{1}\right)}{\partial t}=\theta k_{att}C-k_{\det}\rho_{b}S_{1}$$

(11) $$S_{2}:\rho_{b}\frac{\partial \left(S_{2}\right)}{\partial t}=\theta k_{str}\psi_{t}C$$

where θ represents the volumetric water content; C represents the concentration of NBC in the liquid phase; t represents time; $\rho_{b}$ represents the bulk density of the porous media; x is the distance in the vertical direction; D represents the dispersion coefficient; q represents the Darcy velocity; and $S_{1}$ and $S_{2}$ represent the concentrations of BC nanoparticles retained at site 1 and site 2, respectively. $k_{att}$ and $k_{\det}$ represent the first-order adsorption rate constant (sedimentation rate constant) and desorption rate constant at site 1, respectively; $k_{str}$ represents the first-order exclusion coefficient (sedimentation rate constant) at site 2; and $\psi_{t}$ is a dimensionless constant representing the blocking function.

When the blocking function is of the Langmuir type,

(12) $$\psi_{t}=1-\frac{S_{2}}{S_{\max2}}$$

When the blocking function is depth-dependent,

(13) $$\psi_{t}={\left(\frac{d_{50}+z}{d_{50}}\right)}^{-\beta }$$

where $S_{\max2}$ is the maximum retention amount of NBC at site 2; $d_{50}$ is the average diameter of the soil medium; and β is an empirical parameter that determines the retention curve of NBC in the soil.

When using Hydrus-1D software for NBC breakthrough curve fitting, the upper and lower boundary conditions of the model were set as “fixed flux”. The “two-point kinetic attachment-detachment model” was chosen. Based on the Levenberg–Marquardt least squares method, continuous inversion iterations and fitting corrections were performed to determine the main migration parameters of NBC in the soil column: $k_{att}$, $k_{\det}$, $k_{str}$, and $S_{\max2}$. Among them, the adsorption rate constant ($k_{att}$) and the detachment rate constant ($k_{\det}$) at site 1 reflect the reversible retention capacity of NBC at this point. The sedimentation rate constant ($k_{str}$) and the maximum retention capacity ($S_{\max2}$) at site 2 reflect the irreversible retention capacity of NBC on heterogeneous sites of soil particles.

## 3. Results

### 3.1. Characterization of Nano-Biochar and Soil Properties

The essential physicochemical attributes of BC are presented in Table 2. NBC exhibited an average particle size of 93.81 ± 5.40 nm, while BC possessed a larger average particle size of 11.00 ± 0.80 μm. Notably, the specific surface area of NBC (250 m^2^ g^−1^) surpassed that of BC (140 m^2^ g^−1^) by approximately two-fold, and the pore diameter (pore volume) of NBC also underwent an increase from 6.42 nm (0.06 cm^3^ g^−1^) in BC to 10.5 nm (0.13 cm^3^ g^−1^). Additionally, the *CEC* of NBC and BC was recorded as 231 *cmol*(+)kg^−1^ and 119 *cmol*(+)kg^−1^, respectively. Elemental analysis revealed that NBC and BC were predominantly composed of carbon C, accounting for 75% and 78%, respectively, followed by O content (2.21% and 2.65%, respectively). Both materials exhibited a nitrogen content of less than 1%.

In addition, the ash content results showed no significant difference between the two (9.09%, 9.33%). The level of ash content can reflect its pollution capacity to the environment. In this study, both NBC and BC had low ash content, indicating that they had less impact on environmental pollution. The results of zeta potential measurements for NBC and BC showed that BC exhibited a high negative potential, indicating a large number of negative charges on the surface of BC. Additionally, the absolute value of the zeta potential for NBC (−28.10 ± 0.52 mV) was higher than that for BC (−44.7 ± 0.19 mV), which was due to the presence of more reactive sites and functional group types on the surface of NBC, thus increasing the number of negative charges. The positive or negative nature and magnitude of the zeta potential not only determine the aggregation ability of the particles themselves, but also affect their stability in various media.

**Figure 4:**
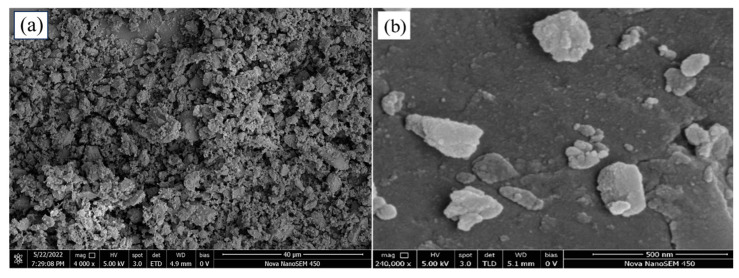
The scanning electron microscopy images of bulk biochar (**a**) and nano-biochar (**b**).

The field emission scanning electron microscopy images of BC and NBC (Figure 4) showed that the bulk BC mainly exhibited micrometer-sized continuous honeycomb-like and layered large particles. The honeycomb structure formed as a result of high-temperature pyrolysis of the BC under anaerobic conditions. This process caused the volatilization of a large amount of substances, thus giving the BC a honeycomb-like appearance both internally and on the surface. In contrast, NBC exhibited a dispersed irregular layered and sheet-like structure, with particle sizes at the nanoscale, and its morphological structure was significantly different from that of BC. The layered and sheet-like structures were caused by continuous collisions and friction between the BC particles, the jar, and the balls throughout the ball milling procedure.

**Figure 5:**
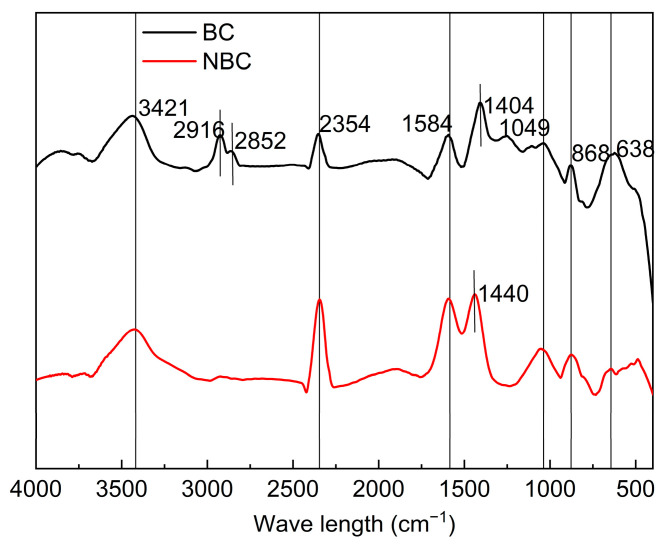
FTIR spectra of bulk biochar and nano-biochar.

As shown in Figure 5, the FTIR spectra of BC and NBC indicate that the BC spectrum exhibits weak aromatic C-H out-of-plane bending vibrations (CO_3_^2−^) in the fingerprint region (638 cm^−1^, 868 cm^−1^), suggesting the presence of a certain amount of carbonates in the BC. The functional group types of NBC are largely consistent with BC. However, NBC exhibits a disappearance of peaks at 2852 and 2916 cm^−1^, indicating a reduction in the number of aliphatic alkyl chains. The peak at 1400 cm^−1^ is absent in NBC, but a C=C skeletal vibration peak appears at 1440 cm^−1^, characteristic of aromatic hydrocarbons. The NBC spectrum also exhibits more pronounced vibrational peaks from aromatic and heterocyclic compounds such as pyridine in the fingerprint region (400–900 cm^−1^), indicating a highly aromatic and heterocyclic structure. This structure favors the occurrence of cation −π interactions in NBC, thereby increasing its surface reaction sites. Additionally, the size of the absorption peaks represents the quantity of each functional group, and the spectra reveal slight differences in the content of surface functional groups between BC and NBC. For instance, the strong peak at 1584 cm^−1^ indicates an increased content of oxygen-containing functional groups (COO-) in NBC compared to the bulk BC.

The basic physicochemical properties of the soil media are shown in Table 3. The two soil samples from Yulin were sandy loam and loam, respectively, both with high sand content and alkaline pH (9.64 and 9.18). The bulk densities were 1.55 and 1.42 g cm^−3^, respectively. The saturated hydraulic conductivities were 95.34 and 60.95 cm d^−1^, respectively. The soil from Changwu was weakly alkaline (pH = 7.94) with a high silt content. The soil type was silt loam with a bulk density of 1.27 g cm^−3^. The saturated hydraulic conductivity (33.89 cm d^−1^) was lower than the other two types of soil. The *CEC* of the three soil types is 89.35, 108.99, and 112.61 mmol(+)kg^−1^, respectively. The Zeta potential results of each soil were −30.9, −26.0, and −20.5 mV, respectively. Affected by local fertilization, precipitation, and other factors, there were significant differences in nitrate nitrogen content among the three types of soil, which were 22.87, 0.91, and 2.54 mg L^−1^, respectively. However, the ammonium nitrogen content was zero in all three soils, which may be related to nitrification in the soil.

**Figure 6:**
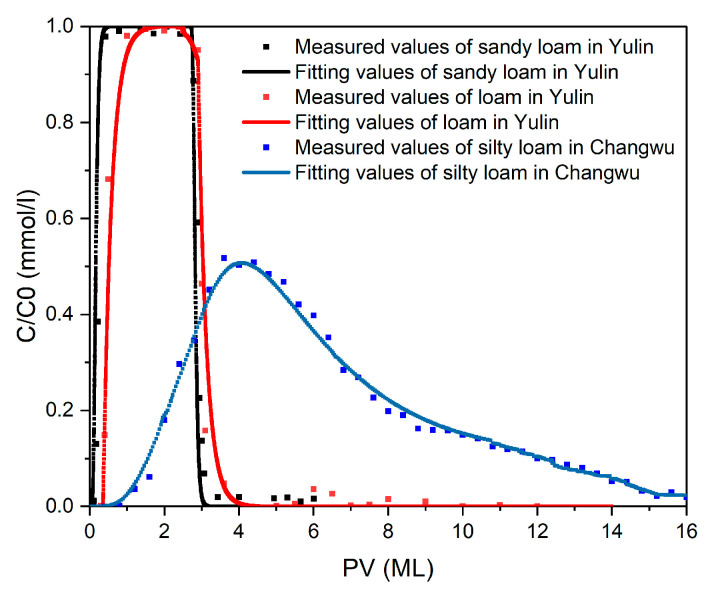
Br^-^ penetration curves and fitting results.

**Figure A1:**
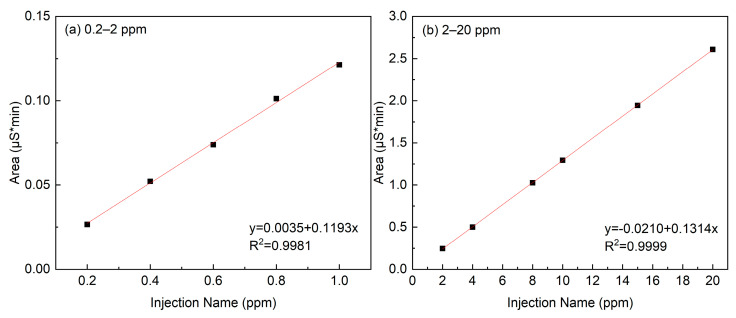
The calibration curves for Br detection of (**a**) 0.2–2 ppm and (**b**) 2–20 ppm.

The disparities in dispersion coefficients among the soil columns primarily stemmed from variations in soil texture, given the uniform pretreatment applied across all columns. The fitting curves of the tracer test are shown in Figure 6 and Figure A1. The fitting parameters showed that the average dispersion coefficients of the soil columns filled with sandy loam, loam, and silt loam were 0.19 ± 0.02, 0.21 ± 0.06 and 0.25 ± 0.05 cm^2^ min^−1^, respectively.

### 3.2. Migration Pattern of Nano-Biochar

**Figure 7:**
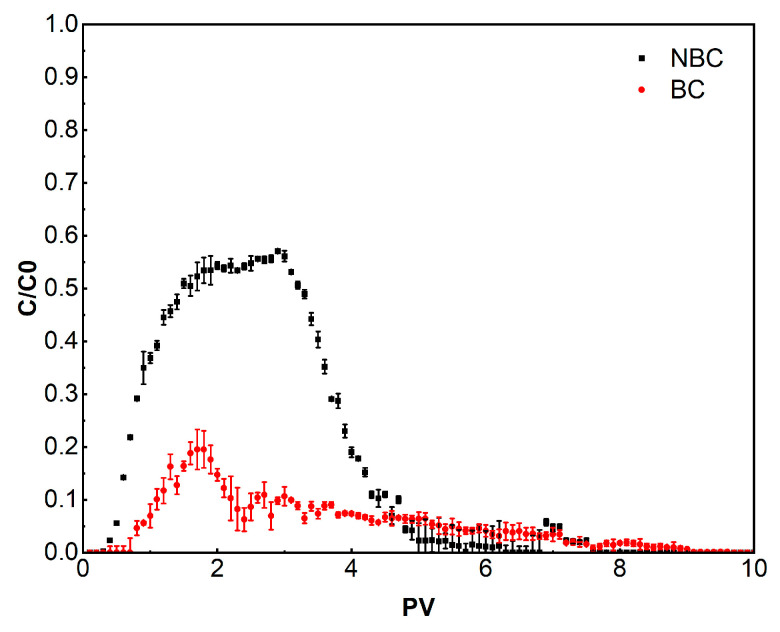
Penetration curves of biochar with different particle sizes.

Compared to the bulk BC, NBC exhibited a stronger migration ability (Figure 7). BC gradually penetrated the soil column near 1 PV, with a maximum relative penetration concentration (C/C_0_) (hereinafter referred to as peak concentration) of 0.20 and exhibited a “tailing” phenomenon. The concentration of BC could not be detected until around 10 PVs. However, NBC began to penetrate the soil column nearly 0.7 PVs earlier than BC, with a peak concentration of 0.57 (C/C_0_), and the penetration ended at around 6 PVs. The overall penetration period of NBC was shortened by approximately 3.7 PVs compared to BC. Additionally, the mass recovery rate of NBC (55.73%) significantly surpassed that of BC (17.17%) (Table 4), more than doubling it. This suggests the majority of the BC was retained in the soil column, while more than half of the NBC effectively penetrated and flowed out of the soil column.

**Figure 8:**
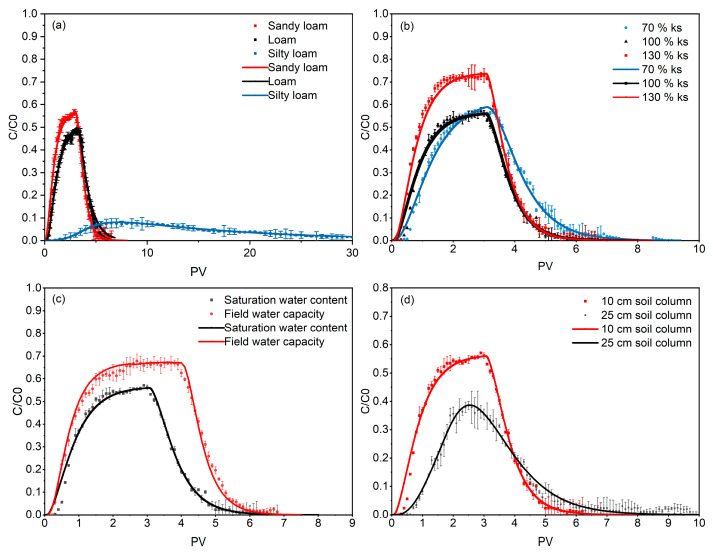
Migration penetration curves and fitting results of nano-biochar in soil under different soil properties (**a**), injection flow rates in sandy loam (**b**), different initial water contents in sandy loam (**c**) and soil column lengths in sandy loam (**d**).

**Figure 9:**
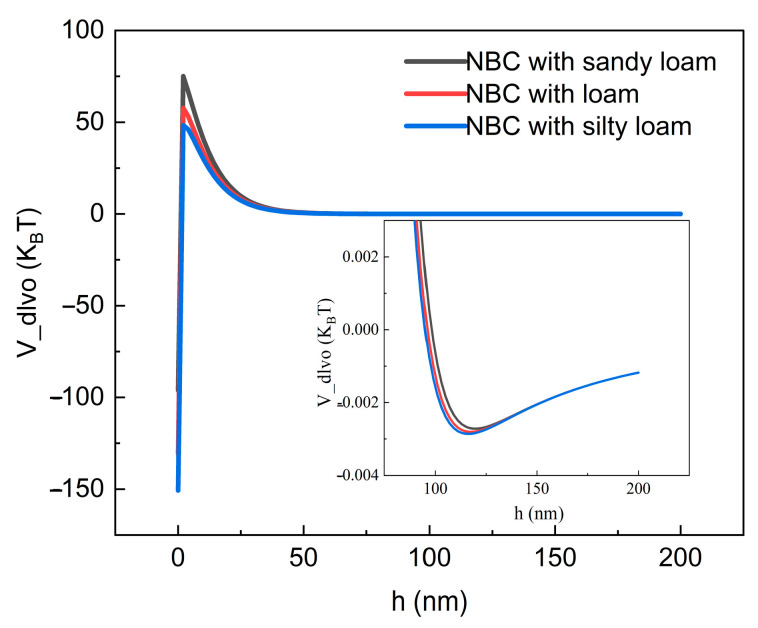
Potential energy of DLVO interaction between nano-biochar and soil of different textures.

Figure 8a shows the migration and penetration curves of NBC in different textural soils. In sandy loam and loam, NBC exhibited similar migration patterns, with a fast penetration rate, high penetration concentration, and short penetration cycle (5.3 PVs, 6.2 PVs). Specifically, in sandy loam, the migration ability of NBC was slightly stronger than in loam. The peak concentration increased from 0.48 in loam to 0.57, and the mass recovery rate also increased from 50.00% to 55.73% (Table 4). In contrast, in silt loam, NBC exhibited a lower penetration concentration, with a peak concentration of only 0.08, but the penetration cycle was prolonged, and trace concentrations of carbon could still be detected at 30 PVs. Due to the long migration cycle, the mass recovery rate in silt loam was also relatively high, reaching 50.98%. The calculation results of DLVO interaction energy for the three types of soil textures in this study showed that as the soil texture changed from fine to coarse, the second minimum energy (*Φ_min_*_2_) between the NBC and soil particles was −0.0029, −0.0028, and −0.0027 K_B_T, respectively (Table 5). The distances between particles (h) when this potential energy was generated were 115.17, 117.19, and 119.21 nm, respectively, with no significant differences. This indicated that the retention capacity of NBC at the secondary potential well was basically the same in the three types of soil. The calculation results of the maximum potential energy revealed that the main interaction energy affecting the migration of NBC was its “highest energy barrier” interaction with the soil particle system; in sandy loam, loam, and silt loam, the maximum repulsive force (*Φ_max_*) that hindered the retention of NBC at the primary potential well decreased significantly in the following order: 75.08, 57.74, and 37.33 K_B_T. This confirmed that as the soil texture changed from coarse to fine, the retention capacity of NBC in the soil increased while its migration ability decreased significantly (Figure 9).

In the same soil texture, the migration ability of NBC was influenced by flow rate, water content, and soil column depth. In the sandy loam, the penetration curves of NBC after adjusting the injection flow rate to 70%, 100%, and 130% of the saturated hydraulic conductivity (Ks) are shown in Figure 8b. The results indicated that the injection flow rate had a certain degree of influence on the migration ability of NBC. As the flow rate increased, the maximum injection concentrations were 0.57, 0.57, and 0.73, with corresponding mass recovery rates of 60.35%, 55.73%, and 74.90%, respectively (Table 4). Under lower flow rate conditions, although the migration amount of NBC did not change much, its penetration time was affected, and the start and end times of NBC penetration under 100% KS were earlier than those under 70% KS. When the flow rate was increased to 130% KS, the penetration period was basically the same as that at 100% KS, but the penetration amount increased by nearly 20%. Differences in initial soil moisture content also altered the migration ability of NBC (Figure 8c). Compared to the soil column in a saturated state, under field capacity conditions, the maximum penetration concentration of NBC increased from 0.57 to 0.68, the time point for the decrease in penetration concentration was delayed from 2.9 PV to 4 PV, and the mass recovery rate also increased from 55.73% to 86.69% (Table 4). This indicates that the migration ability of NBC under field capacity conditions is stronger than that under saturated moisture content conditions. When the length of the soil column was increased to 25 cm, the migration ability of NBC was significantly reduced compared to the 10 cm soil column (Figure 8d), with a slower penetration rate and a decreased maximum penetration concentration of 0.39. The mass recovery rate (36.93%) was approximately 10% lower than that of the 10 cm soil column (Table 4). This suggests that the deeper soil layer imposed increased retardation effects on the migration of NBC.

### 3.3. Migration Model of Nano-Biochar

The two-site kinetic model was used to fit the penetration curves of NBC under different variables, and the fitting curves are shown in Figure 6. The basic model parameters for fitting are listed in Table 6. Except for silt loam, which used the depth blocking function (M) due to its poor fitting effect, the curves under other variables were fitted using the Langmuir-type blocking function (L). The goodness of fit (R^2^) between the simulation results and the measured values was above 98% (Table 6), indicating that the simulation results match the real migration behavior of NBC in soil. To further explore the migration patterns and mechanisms of NBC in soil under different variables, comparing the fitting parameters ($k_{att}$, $k_{\det}$, $k_{str}$, and $S_{\max2}$) is of critical significance. Among them, the NBC at site 1 was a reversible retention site, and $k_{att}$ and $k_{\det}$ reflected the desorption rate and deposition rate of NBC at site 1, while the deposition rate constant ($k_{str}$) and maximum retention capacity ($S_{\max2}$) at site 2 reflected the irreversible retention capacity of NBC on heterogeneous sites of soil particles.

The fitting parameters of the model showed a good response to the migration ability of NBC in different soil textures. With the increase in sand content in the soil, $k_{att}$/$k_{\det}$ at site 1 decreased successively, with values of 29.37, 1.97, and 1.32 min^−1^, respectively. At site 2, $k_{str}$ was 0.99, 0.009, and 0.0069 min^−1^, respectively, showing a significant decreasing trend. Silt loam showed significant differences in fitting parameters compared with other soils, confirming that silt loam has a stronger retention capacity for NBC, which is consistent with the results of the penetration curves. When the soil texture changed from loam to sandy loam, $k_{att}$, $k_{\det}$, $k_{str}$ and $S_{\max2}$ all decreased to some extent, with $S_{\max2}$ decreasing from 600 mg g^−1^ to 225 mg g^−1^. This indicates that loam has a stronger migration hindrance to NBC than sandy loam, regardless of whether it is at site 1 or site 2.

With the increase in NBC injection flow rate, the ratio of $k_{att}$ to $k_{\det}$ at site 1 gradually decreased. When the flow rate was 130% Ks, the ratio was close to 1, indicating that under this condition, the adsorption and desorption rates of NBC at site 1 had approached equilibrium. The value of $S_{\max2}$ at site 2 increased with the flow rate, with values of 432, 225, and 125 mg g^−1^, respectively, indicating that high flow rates hindered the retention capacity of NBC in soil. However, the value of $k_{str}$ did not show a significant pattern with the flow rate, which may be due to its small value, already in the order of 10^−2^ to 10^−3^, making it difficult to reflect differences.

Under two conditions, namely adjusting the water content from saturation to field capacity and increasing the soil column length from 10 cm to 25 cm, the ratio of $k_{att}$/$k_{\det}$ and $S_{\max2}$ both showed a decreasing trend in the former case, consistent with the results shown by the penetration curves. This indicated that the initial water content at field capacity was more conducive to the migration of NBC than at saturation. However, the latter condition exhibited an opposite trend, with NBC being more likely to be retained in the 25 cm soil column.

## 4. Discussion

### 4.1. The Influence of Intrinsic Physicochemical Properties on the Migration of NBC

NBC exhibited a stronger migration ability compared with traditional BC, which may be attributed to the following two aspects. (1) Particle size difference: the movement of particles in porous media is affected by the particle size itself and the pore size of the media. When encountering pores with diameters smaller than the particles, exclusion effects may occur, causing the particles to be mechanically retarded outside the small pores [43,44]. Therefore, NBC is more prone to migration, due to its smaller particle size, while micrometer-sized bulk BC is more susceptible to exclusion effects, leading to retention in the media. (2) Surface charge difference: compared to the bulk BC, NBC has a higher negative Zeta potential (Table 2). Therefore, when interacting with soil media, the double-layer repulsive potential energy between NBC and the soil matrix (Table 3), which also has a negative potential, is greater. That is, NBC with a higher negative Zeta potential moving in the soil experiences stronger repulsion between its surface negative charge and the negative charge of the soil matrix [25,45]. This keeps NBC always in the center of pores with higher flow velocities. This hydrodynamic effect allows nanoparticles to have a higher flow velocity than the carrier matrix, resulting in faster and stronger migration rates [24,25]. In addition, studies have shown that particles (BC) with lower absolute Zeta potential values (within 30 mV) tend to agglomerate and are more likely to form large aggregates after entering the soil media [46,47,48], thereby triggering exclusion effects and leading to particle retention.

### 4.2. The Influence of External Transport Surroundings on the Migration of NBC

The results indicate that there are significant differences in the migration ability of NBC in soils with different textures, and its migration ability increases significantly as the soil texture changes from fine to coarse. This is mainly due to the fact that the migration of nanoparticles is not only affected by their own characteristics but is also closely related to the physicochemical properties of porous media: in finer-textured soils, the proportion of small particle size particles and small pores is relatively high [49], and the permeability is also smaller. Since the diffusion coefficient of nanoparticles is small, they are not easy to diffuse into small pores with low concentrations and slow flow rates. Therefore, consistent with previous studies, the results of this study show that the ability of nanoparticles to migrate downward is relatively weak in fine-textured soils (silt loam), while the migration ability of NBC gradually increases in loam and sandy loam [26,50,51]. In addition, soil properties such as pH, *CEC*, and negative Zeta potential can alter the interaction energy and adsorption capacity between particle systems. Relevant studies have shown that the migration ability of nanoparticles is positively correlated with the above soil properties [50], which is consistent with the results of this study on the physicochemical properties of the three soil textures and the migration patterns of NBC. At the same time, the mechanism can be further revealed from the DLVO theory: the retention of nanoparticles in porous media is affected by secondary potential well effects and the highest energy barrier [25,33,34]. The former reflects whether the weak attractive energy (second minimum energy) in the DLVO theory is large enough to attract a certain number of particles to stay in the secondary potential well. The latter represents the maximum repulsive force (highest potential energy) in the DLVO theory that hinders the retention of particles at the primary potential well. When the attractive force of the system is small and the repulsive force is large, particles are less likely to be retained and more prone to migration. This is consistent with the calculation results of DLVO interaction energy for the three soil textures in this study, that is, the interaction energy gradually increases as the texture changes from fine to coarse, thereby affecting the migration ability of NBC.

The migration ability of NBC in the same soil texture is also influenced by factors such as injection flow rate, initial soil water content, and soil column depth to varying degrees. Studies have shown that the hydraulic properties of porous media (water content, pore water flow rate) have a significant impact on the migration of nanoparticles. When the porous media is in an unsaturated state, nanoparticles are likely to be retained at the solid–liquid–gas triple interface, resulting in reduced mobility [52,53,54]. Notably, contrary to previous research findings, this study found that the migration ability of nanoparticles is significantly stronger in an unsaturated state than in a saturated state. This phenomenon may be due to the large number of large pores and good connectivity in the filled sandy loam soil column. Combined with the size exclusion effect, the migration of nanoparticles in the unsaturated state is more likely to be restricted to large pores, increasing the probability of preferential flow formation and even leading to nanoparticle transport rates exceeding pore water flow rates [55,56,57]. In addition, changes in injection flow rate may alter the velocity distribution of pore water, thereby affecting particle transport in pore water [58]. As the injection flow rate increases, on the one hand, convection becomes dominant in the migration of nanoparticles, and NBC moves along with the solution, reducing the frequency of collisions between particles and the surface of the porous media [59]. This, in turn, reduces the probability of nanoparticle adsorption [31,60], enhancing their mobility. On the other hand, increased flow shear forces at high flow rates may lead to the desorption of already adsorbed particles [61]. Therefore, the results of this study show that when the injection flow rate is increased to 130% KS, the migration ability of NBC is significantly enhanced, while there is no significant change in migration ability at 70% and 100% KS. This may be due to the dominant role of adsorption forces between particle systems at relatively lower flow rates [32,62]. Regarding different soil column depths, compared to the 10 cm soil column, the mobility of NBC in the 25 cm soil column is significantly reduced. This indicates that factors such as nanoparticle aggregation, exclusion effects, and soil surface roughness during migration affect the retention of biochar nanoparticles in the soil column. As the depth of the soil column increases, the retention of nanoparticles in the soil column increases exponentially [24,25], leading to a reduction in the outflow of nanoparticles at the outlet and a corresponding decrease in overall migration ability.

## 5. Conclusions

This study focuses on NBC prepared through the pyrolysis of apple tree branches followed by ball milling. Using indoor soil column migration experiments, a systematic and thorough exploration of the migration patterns of BC in soil under different factors such as BC particle size, soil texture, injection flow rate, initial soil water content, and soil depth was conducted. Additionally, analytical methods such as particle system characteristics, the DLVO theory, and two-point kinetic models were employed to quantitatively and qualitatively reveal the migration mechanisms of NBC. The main conclusions indicate that NBC exhibits a stronger soil migration ability than traditional BC due to its small particle size and surface charge characteristics. The migration performance is affected by soil texture, water content, flow velocity, and depth. Migration ability is weakened in fine soil, but enhanced under unsaturated and high flow velocity conditions. The aggregation and exclusion effects of deep soil layers cause NBC to mainly remain in the surface layer, reducing its migration ability. The two-point kinetic model can be used to simulate NBC migration, providing a basis for predicting behavior in complex environments. Therefore, when using NBC for restoration, it is necessary to consider its migration characteristics under different environmental conditions.

## Figures and Tables

**Table 1:** Basic information on soil column migration experiments.

Number	Initial Water Content	Soil Texture	Injection Velocity	Biochar Type	Soil Column Length cm	Pore Volume mL
1	Saturated water content	Silty loam	100% KS	NBC	10	394.8 ± 23.6
2	Saturated water content	loam	100% KS	NBC	10	433.2 ± 18.4
3	Saturated water content	Sandy loam	70% KS	NBC	10	548.9 ± 21.8
4	Saturated water content	Sandy loam	100% KS	NBC	10	541.0 ± 13.2
5	Saturated water content	Sandy loam	130% KS	NBC	10	568.4 ± 7.5
6	Saturated water content	Sandy loam	100% KS	BC	10	551.8 ± 16.1
7	Saturated water content	Sandy loam	100% KS	NBC	25	1041.3 ± 30.4
8	Field capacity	Sandy loam	100% KS	NBC	10	551.6 ± 10.5

Note: Each experimental group was repeated three times.

**Table 2:** Basic physicochemical properties of biochar particles.

Properties	BC	NBC
Particle size (μm)	11.00 ± 0.80	0.093 ± 0.0054
Specific surface area (m^2^ g^−1^)	140 ± 0.27	250 ± 0.14
Pore size (nm)	6.42 ± 0.16	10.5 ± 0.110
Pore volume (cm^3^ g^−1^)	0.06 ± 0.01	0.13 ± 0.01
Cation exchange capacity (*cmol*(+)kg^−1^)	119 ± 0.05	231 ± 0.11
*C/H/O/N* (%)	75.30/2.65/12.30/0.69	78.10/2.21/9.45/0.87
Ash content (%)	9.09 ± 0.31	9.33 ± 0.20
Zeta potential (mV)	−44.7 ± 0.19	−28.10 ± 0.52

**Table 3:** Basic physicochemical properties of soil media.

Soil Type	Sandy Loam	Loam	Silty Loam
Soil texture (%)	Clay (1.36)/Silt (19.09)/Sand (79.55)	Clay (3.12)/Silt (36.51)/Sand (60.37)	Clay (8.05)/Silt (69.73)/ Sand (22.22)
pH	9.64 ± 0.11	9.18 ± 0.04	7.94 ± 0.06
Soil bulk density (g cm^−3^)	1.55 ± 0.02	1.42 ± 0.05	1.27 ± 0.03
Saturated water conductivity (cm d^−1^)	95.34 ± 0.18	60.95 ± 0.16	33.89 ± 0.13
Cation exchange capacity (mmol(+)kg^−1^)	89.35 ± 0.12	108.99 ± 0.06	112.61 ± 0.08
Zeta potential (mV)	−30.9 ± 0.47	−26.0 ± 0.26	−20.5 ± 0.11
NO_3_^−^-N content (mg L^−1^)	22.87 ± 0.02	0.91 ± 0.02	2.54 ± 0.03
NH_4_^+^-N content (mg L^−1^)	0.00	0.00	0.00

**Table 4:** Mass recovery of biochar in outflows under different conditions.

Number	Type	Variable	Mass Recovery (%)
4	Particle size	NBC	55.73 ± 1.21 a
6		BC	17.17 ± 1.89 b
4	Soil texture	Sandy loam	55.73 ± 1.31 a
2		Loam	50.00 ± 1.52 b
1		Silty loam	50.98 ± 2.38 b
3	Injection velocity	70% Ks	60.35 ± 0.95 b
4		100% Ks	55.73 ± 1.17 a
5		130% Ks	74.90 ± 2.01 c
4	Initial water content of soil column	Saturated water content	55.73 ± 1.21 b
8		Field capacity	86.69 ± 2.78 a
4	Soil column depth	10 cm	55.73 ± 1.21 a
7		25 cm	36.93 ± 3.00 b

Note: Different lowercase letters indicate significant differences (*p* < 0.05) in mass recovery of biochar between variables under different experimental types.

**Table 5:** Main parameters of DLVO interaction potential energy.

Soil Type	*Φ_max_* ^a^ (K_B_T)	*Φ_min_*_2_ ^b^ (K_B_T)	*h* ^c^ nm
Sandy loam	75.08	−0.0027	119.21
loam	57.74	−0.0028	117.19
Silty loam	37.33	−0.0029	115.17

Note: a, The highest potential energy between biochar nanoparticles and soil particles; b, the second minimum potential energy; and c, the distance between biochar nanoparticles and soil particles at which the second minimum potential energy occurs.

**Table 6:** Nano-biochar migration model simulation parameters under each variable.

Number	Blocking Function	S_max2_ Mg g^−1^	k_str_ min^−1^	k_att_ min^−1^	k_det_ min^−1^	k_att_/k_det_	R^2^
1	M	\	0.999	1.298	0.044	29.37	0.989
2	L	600	0.009	2.493	1.265	1.97	0.987
3	L	432	0.007	0.162	0.068	2.38	0.993
4	L	225	0.012	0.118	0.090	1.32	0.993
5	L	125	0.008	0.129	0.130	0.99	0.996
6	\	\	\	\	\		
7	L	1000	0.003	0.071	0.029	2.47	0.989
8	L	500	0.008	0.079	0.200	0.40	0.994

## Data Availability

Data are contained within the article.
